# The New Independence; Made in America; Patriotism in the Medical Profession

**Published:** 1915-04

**Authors:** G. Henri Bogart

**Affiliations:** Paris, Illinois


### The New Independence; Made in America; Patriotism in the Medical Profession.

DR. G. HENRI BOGART, PARIS, ILLINOIS.

When the great war first commenced the medical man wondered where he was to obtain the customary drugs; now the restrictions of commerce has put the hobble on the other foot and the hemmed in belligerents—and lots of other folks—are wondering about the food supply.

The whole world has been crying for the moon, has been turning to the absent distance for the glamor, passing up the "something just as good" or better at home.

The Martians may be supposed to gaze down at their evening star, our earth, in its wondrous purity of white beauty, unthinking of the bottomless, spring-thawed roads of Illinois, or the discoveries of the vice commissions, blotches on its silvery sheen, for that star is our earth, and other drawbacks.

I have ridden through the woodlands of Indiana, when the hillsides glowed with acres of the purple splendor of the digitalis, while the farmed cursed it, the stagger weed that poisoned his cattle, or compelled him to forego the use of the lush pasturage, and then stopped at the drug store to purchase digitalin "made in Germany." I have seen the village physician paying a laborer to grub the "Wandering Milkweed" from his pasture lot, and at the same time prescribing the alkaloid from those same laboratories across the sea, the while his father had learned the virtues of pleurisy root from the older botanic school.

No, I do not mean that we shall go back, and with shears and shovel gather our sources for infusion and decoction.

America has splendid laboratories with well won reputation for alkaloids, specific tinctures and extracts second to none; we have unlimited fields of crude material, the plants and minerals in abundance, either neglected or harvested for shipment to the laboratories across the ocean, on whom we have come to depend.

We prate about our independence, and then depend, slavishly, upon those others, while neglecting our own manufacturers. We cannot expect our own chemists to flourish unless we support them, for we read of that which grows by that it doth feed upon.

I have trellises of roses about my home, vines that yield a wealth of perfumed beauty, that would not be, did I not furnish the support and loving care for their growing.

As a duty, as pure patriotism, we should declare a new era of independence and we need not wait until the Fourth of July, either, rather

"Act, act in the living present."

We need no Congress, no Liberty Hall; we need just you, and you, and you, all pulling together for the sustenance of home remedies.

I have been amused and disgusted at the prominence given the "Twilight Sleep," with all the twaddle and rigmarole of the lay press, exploiting that something new that drew the Greeks about St. Paul, on Mars Hill, when right here in America we have had for years, a more dependable preparation, in tablet form on the market, permanently dispensed, and with an abundant free literature on the subject, and nothing has been said about it, only in a business way to the profession; you could not have driven a newspaper or a magazine reporter to have peeped through the portals of the possibilities of this "Made in America" release from the pangs and terrors of childbirth, for the story would have been commercial, would have lacked the twang and novelty of importation, and would have violated the ethics of publication, by advertising something as a news feature. This publication, with gratis literature has been before the American public, but met silence, while its more clumsy foreign substitute was boomed as a sensation, a scoop.

There is the axiom of supply and demand that answers the question as to the ability of our chemists and pharmacists to supply all the skill and products we need.

We spend many millions, annually, on scientific education, and then calmly close our eyes and leave the men and women whom we have trained, to potter and piddle, for want of a demand for their services, that shall sharpen their faculties and reward their efforts; throttling their ambitions instead of spurring them on.

Here is one example of what incentive will do.

Edison was using tons of carbolic acid each week, when the war stopped his supply. He had been told that our coals would not furnish it. He did not shut down his works; he investigated the making of a synthetic product, got busy, and within a month was making a superior product, cheaper than the foreign chemist had produced it.

America can furnish as good and better chemicals than can the foreigner if the rank and file support the demand. Our chemists and pharmacists are able, willing and anxious to furnish for our utmost needs if we give the opportunity. If you are not satisfied with the reactions of a remedy; if you need something that it lacks, tell your manufacturer, write him candidly, take him into your confidence; he is more than willing to co-operate.

If you know what you want, he will be more than willing to meet you more than half way, to know and to solve your difficulties.

Be patriotic in the highest sense, that of the upbuilding of your own country, which is better than hurting and destroying your neighbor nation.

Make yourself one of the signers of the new Declaration of Independence and do it now; having done it, keep on doing it.

